# 

**Published:** 1897-06

**Authors:** 


					﻿CONTENTS.
ORIGINAL ARTICLES—
Total Abdominal Hysterectomy............................... 263
Some remarks on the Treatment of Chronic Otorrhcea........ 265
Some of the Evils that Threaten the Medical Profession.... 271
SELECTIONS AND ABSTRACTS—
Earache: Iter Significance and Early Management............ 275
The Various Discharges of the Male Urethra................. 280
The Treatment of Varicose Veins........................... 282
Sage in Hyperidrosis....................................   283
Chronic Interstitial Nephritis in Childhood..............  284
Treatment of Rupture of the Kidney........................ 285
Care of Teeth of Children....,..........................   286
Merycism............................................. "...	287
Recent Advances in General Medicine.... .................  290
Treatment of Dysmenorrhea with the Galvanic Current....... 291
The Significance of Sudden Acute Abdominal Pain........-... 292
Blennorrhea Treated with Ichthyol.......,...............   294
Hydrastis Canadensis in the Treatment of Bronchial Catarrh....	295
Successful Laparotomy in the First Hour of Life............ 296
Helminthiasis Extraordinary............ ..............4....	296
A Simple and Practical Method of Sterilizing Soft-Rubber
Catheters .............................................. 297
Tonsillar Cough..........................................  299
Doctors’ Bills Must be Paid............................... 299
The Degree of Anesthesia which Should be Induced Prior to Sur-
gical Operations................................ • • • •	300
Newspaper Attack on the Code of Ethics.............	301
Operative Treatment of Irreducible Dislocation of the Shoulder,
Recent or Old, Simple or Complicated..............  304
A New Method for Localizing Brain Lesions.........   305
A Case of Castration for Hypertrophied Prostate..... 306
A Remedy in Nervous Disorders when Characterized by Melan-
cholia....................................  •	• ... 306
Boiled Milk...................................       307
SOCIETY REPORTS—
Richmond Academy of Medicine and Surgery.......... 308
EDITORIAL...........................................   310
BUSINESS DEPARTMENT..................................  312
PRESCRIPTIONS.......................................   315
				

